# Peptide Nucleic Acid Knockdown and Intra-host Cell Complementation of *Ehrlichia* Type IV Secretion System Effector

**DOI:** 10.3389/fcimb.2017.00228

**Published:** 2017-06-07

**Authors:** Pratibha Sharma, Omid Teymournejad, Yasuko Rikihisa

**Affiliations:** Department of Veterinary Biosciences, Ohio State UniversityColumbus, OH, United States

**Keywords:** PNA, *Ehrlichia chaffeensis*, type IV secretion, complementation, autophagy, effector, Etf-1

## Abstract

Survival of *Ehrlichia chaffeensis* depends on obligatory intracellular infection. *One* of the barriers to *E. chaffeensis* research progress has been the inability, using conventional techniques, to generate knock-out mutants for genes essential for intracellular infection. This study examined the use of Peptide Nucleic Acids (PNAs) technology to interrupt type IV secretion system (T4SS) effector protein expression in *E. chaffeensis* followed by intracellular complementation of the effector to determine its requirement for infection. Successful *E. chaffeensis* infection depends on the *E. chaffeensis*-specific T4SS protein effector, ehrlichial translocated factor-1 (Etf-1), which induces Rab5-regulated autophagy to provide host cytosolic nutrients required for *E. chaffeensis* proliferation. Etf-1 is also imported by host cell mitochondria where it inhibits host cell apoptosis to prolong its infection. We designed a PNA specific to Etf-1 and showed that the PNA bound to the target region of single-stranded Etf-1 RNA using a competitive binding assay. Electroporation of *E. chaffeensis* with this PNA significantly reduced Etf-1 mRNA and protein, and the bacteria's ability to induce host cell autophagy and infect host cells. Etf-1 PNA-mediated inhibition of ehrlichial Etf-1 expression and *E. chaffeensis* infection could be intracellularly trans-complemented by ectopic expression of Etf-1-GFP in host cells. These data affirmed the critical role of bacterial T4SS effector in host cell autophagy and *E. chaffeensis* infection, and demonstrated the use of PNA to analyze the gene functions of obligate intracellular bacteria.

## Introduction

Discovered in 1986 and designated as a nationally notifiable disease in 1998 by the CDC, human monocytic ehrlichiosis (HME) is one of the most prevalent, life-threatening, emerging tick-borne diseases in the US (Paddock and Childs, 2003; CDC, 2015). HME is caused by infection with *Ehrlichia chaffeensis*, an obligatory intracellular bacterium in the order *Rickettsiales* (Paddock and Childs, 2003; Rikihisa, 2015). *E. chaffeensis* replicates within human monocytes-macrophages, and causes severe flu-like symptoms accompanied by hematologic abnormalities and signs of hepatitis. Currently, the only choice of treatment is the broad-spectrum antibiotic doxycycline, which is only effective if initiated early because delayed therapy initiation, for example due to misdiagnosis, can lead to severe complications or death. The presence of underlying illness or injury, stress, immunosuppression, and/or coinfection with other tick-borne pathogens can lead to severe complications or death in 2–5% of infected individuals (Paddock and Childs, 2003). No vaccines exist for HME. Tick-borne diseases have risen dramatically in the past two decades, and continue to rise (Paddock and Yabsley, 2007), underscoring the importance of developing a novel therapeutic approach for infections with tick-borne intracellular bacteria.

Because *E. chaffeensis* has a small genome of 1.176 Mb and relatively few genes encoding proteins needed for biosynthesis and metabolism, it cannot survive outside of eukaryotic host cells and relies heavily on host-derived nutrients for its replication (Rikihisa, 2015). As *E. chaffeensis* cannot survive anywhere else, with adequate knowledge and tools, targeting specific *E. chaffeensis* genes that enable its intracellular survival and proliferation may be ideal for new anti-*Ehrlichia* therapeutic strategies. However, one of the major barriers to *E. chaffeensis* research progress has been the inability to generate *E. chaffeensis* knockout mutants for genes essential for obligatory intracellular infection, using conventional techniques. Thus, it has not been feasible to fulfill molecular Koch's postulates by studying phenotypes of knockout mutants and restoring lost functions by complementation.

*E. chaffeensis* encodes a type IV (type IVa, VirB/D) secretion system (T4SS) that mediates the transport of bacterial DNA and/or proteins, referred to as effectors/substrates, across the bacterial membrane into the eukaryotic cell to deregulate or modulate target cell functions for the benefit of the bacteria (Alvarez-Martinez and Christie, 2009; Rikihisa, 2015). T4SS machinery and effectors are major proteins produced by *E. chaffeensis*, suggesting they merit such extensive energy expenditures by the pathogen (Bao et al., 2009; Lin et al., 2011). *E. chaffeensis* translocated factor 1 (Etf-1) is the first experimentally proven T4SS effector in the genus *Ehrlichia* (Liu et al., 2012). Etf-1 is highly produced and secreted by *E. chaffeensis* in human monocytes (Lin et al., 2011; Liu et al., 2012); neutralization of secreted Etf-1 by delivering anti-Etf-1 IgG into the cytoplasm of host cells inhibited cellular infection by *E. chaffeensis* (Liu et al., 2012), whereas ectopic Etf-1 expression enhanced *E. chaffeensis* infection (Lin et al., 2016). Secreted Etf-1 has two important functions: (1) it localizes to mitochondria and blocks host cell apoptosis, and thereby allows sufficient time for *E. chaffeensis* replication (Liu et al., 2012); and (2) it is required, via a novel protein-protein interaction, for *E. chaffeensis*-induced Rab5-regulated autophagy to make host cytosolic nutrients available for *E. chaffeensis* (Lin et al., 2016). Because Etf-1 regulates host cell functions, Etf-1 influences the entire intracellular bacterial population.

Unlike some facultative intracellular bacteria including the well-studied *Legionella pneumophila*, to date, no T4SS system knockout mutants have been obtained in any member of the order Rickettsiales, and stable targeted mutagenesis has not been achieved in *E. chaffeensis* (Cheng et al., 2013). Despite the recent use of Himar1 transposon random insertion mutagenesis in *E. chaffeensis* (Cheng et al., 2013), genetic manipulation of *E. chaffeensis* must overcome many difficulties, e.g., (1) the lack of resistance markers except for a single antibiotic (spectinomycin/streptomycin; tetracycline cannot be used as it is the only clinically effective antibiotic) limits screening for *E. chaffeensis* transformants; (2) the very slow process for selecting mutants, i.e., can take weeks due to poor transformation efficiencies, viability, and slow mutant bacterial growth (Cheng et al., 2013); and (3) the lack of plasmids or phages capable of replicating in this group of bacteria (family *Anaplasmataceae*).

Peptide Nucleic Acids (PNAs) are single-stranded synthetic nucleic acids with a pseudopeptide backbone in place of the phosphodiester-linked sugar and phosphate found in traditional oligos. PNAs are chemically stable, resistant to enzymatic degradation, with low tissue toxicity (Good and Nielsen, 1998). PNA-DNA binding is stronger and the specificity is higher than DNA-DNA binding (Nielsen and Egholm, 1999). Anti-sense PNAs that disrupt transcription and translation have been developed for gene therapy, genetic disorder diagnostics, infection prophylaxis, and molecular tools for nucleic acid manipulations (Nielsen et al., 1991; Good and Nielsen, 1998; Janowski et al., 2005; Abushahba et al., 2016). The PNA approach is advantageous for working with *E. chaffeensis* because PNAs do not require mutation/selection of bacteria. Furthermore, partial reduction of expression can be achieved allowing study of essential genes, where investigations of a phenotype by a true knockout are not possible. This approach, however, has not been used for obligatory intracellular bacteria, except for *Rickettsia montanensis* and *Rickettsia typhi* (Pelc et al., 2015), and complementation assays have never been accomplished after PNA knock down. In this study, we designed anti-sense PNAs to disrupt the T4SS effector Etf-1, and determined the effectiveness of target gene knockdown, blockage of host cell autophagy induction, and *E. chaffeensis* infection in human cells, and whether Etf-1 inhibition could be trans-complemented within host cells.

## Materials and methods

### Cultivation of *E. chaffeensis* and host cells

*E. chaffeensis* Arkansas (Dawson et al., 1991) was cultured in the human monocytic leukemia cell line THP-1 (ATCC, Manassas, VA) in RPMI 1640 medium (Corning Cellgro, Manassas, VA) supplemented with 5% fetal bovine serum (FBS; Atlanta Biologicals, Lawrenceville, GA) and 2 mM L-glutamine (Invitrogen, Grand Island, NY) at 37°C in 5% CO_2_ and 95% air in a humidified atmosphere as previously described (Barnewall and Rikihisa, 1994). Cells were monitored for 2–3 days for infection using HEMA 3 stain (Thermo Fisher, Waltham, MA) on cytocentrifuged specimens (Cytospin™4 Cytocentrifuge, Thermo Fisher), and passaged/harvested when the percentage of infected cells reached >95% as previously described (Barnewall et al., 1997). The human kidney epithelial cell line HEK293 (ATCC) was cultured in Dulbecco's minimal essential medium (DMEM; Corning Cellgro) supplemented with 10% FBS and 2 mM l-glutamine as previously described (Miura et al., 2011).

### PNA synthesis, biotin labeling, and RNA-PNA hybridization

**Figure 1:**
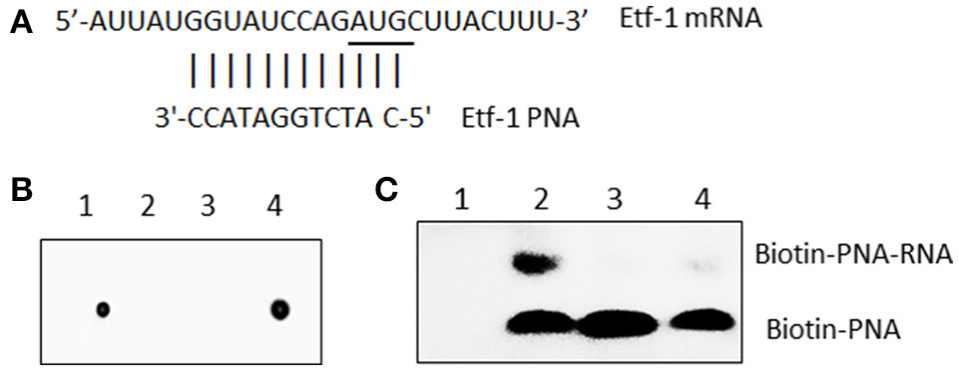
*E. chaffeensis* Etf-1 PNA bound to the target ssRNA. **(A)** Etf-1 PNA targeted the putative Shine-Dalgarno region and translational start site (underline). **(B)** Dot blot analysis of biotin-labeled Etf-1 and streptavidin-HRP-labeled CTL PNA. Dot 1–4: Biotin-CTL PNA, Unlabeled CTL PNA, Unlabeled Etf-1 PNA, and Biotin-Etf-1 PNA. **(C)** Electrophoresis migration shift assay of biotinylated Etf-1 PNA bound to ssRNA. Lane 1, no labeled PNA, lanes 2–4 labeled PNA incubated with 0, 10 ×, and 1 × non-labeled PNA, respectively.

Custom PNA oligomers were synthesized, and PNA quality and quantity were verified by HPLC and MS (PNABio, Newbury Park, CA). An antisense oligomer was designed to the start codon of Etf-1 (Figure 1). A scrambled PNA control (CTL PNA; 3′-CACATATCTCGG-5′) also was designed. To ensure the specificity of the PNA sequence, each sequence was blasted against *E. chaffeensis* Arkansas whole genome (Dunning Hotopp et al., 2006). PNA oligomers were biotinylated using EZ-Link Sulfo-NHS-Biotin according to the manufacturer's instructions (Thermo Fisher). Labeled PNA was purified using Pierce C18 Spin Columns (Thermo Fisher). Dot blots were used to confirm the successful biotinylation of oligomers as previously described (Pelc et al., 2015). The membrane was developed using Pierce Chemiluminescent Nucleic Acid Detection Module Kit (Thermo Fisher) according to the manufacturer's instructions.

To verify that Etf-1 PNA binds to the targeted region, the Etf-1 ssRNA molecule 5′-AUUAU**GGUAUCCAGAUG**CUUACUUU-3′ (bold nucleotides represent the RNA sequence corresponding to the designed PNA sequence, Figure 1) was synthesized by Sigma-Aldrich (St. Louis, MO). RNA-PNA hybridization was performed in a 15 μl reaction mixture with 2.0 μl (200 μM) ssRNA and 10 μM biotin-labeled Etf-1 PNA or 0, 10, and 100 μM of unlabeled PNA with 1 μl of RNaseOUT (Thermo Fisher) in 250 mM Tris buffer (pH 7.2) at room temperature for 30 min. The samples were run on a 2% agarose gel in TBE buffer (40 mM Tris, pH 8.3, 45 mM boric acid, and 1 mM EDTA) and transferred to an Amersham HyBond N+ membrane (GE Healthcare Life Sciences, Marlborough, MA) using a semi-dry blotting apparatus (WEP, Seattle, WA). Membranes were UV cross-linked in a Stratalinker® UV Crosslinker 1800 (Stratagene, San Diego, CA) and developed as above.

### Transfection of host cell-free *E. chaffeensis*

*E. chaffeensis*-infected THP-1 cells (~4 × 10^7^ cells, >90% infected cells from two T75 flasks, ~100 bacteria/cell based on 16S rRNA qPCR) were harvested by centrifugation at 400 × g for 5 min. The pellet was resuspended in 20 ml of 300 mM sucrose and sonicated on ice twice at setting 2 for 5 s using a W-380 Sonicator (Heat Systems-Ultrasonics, Farmingdale, NY). Unbroken cells were removed by centrifugation at 1,000 × g for 5 min. The supernatant was collected after centrifugation at 1,700 × g for 5 min and passed through 2.7- and 5.0-μm GD/X nylon filters (Whatman, Florham Park, NJ) sequentially to remove cell debris, and finally centrifuged at 9,700 × g for 10 min as previously described (Liu et al., 2012). The bacterial pellet was resuspended in 200 μl of ice-cold 300 mM sucrose.

To deliver PNA, 3 μg of Etf-1 or CTL PNA in 10 μl nuclease-free water was mixed with 100 μl of host-cell free bacteria in 300 mM sucrose in a sterile 0.2 cm gap electroporation cuvette (Bio-Rad, Hercules, CA) on ice. *E. chaffeensis* bacteria were electroporated using a Gene PulserXcell Microbial System (Bio-Rad) by applying a ~9 ms pulse (2,000 V, 25 μF, 400 Ω). Immediately after electroporation, 400 μl of prewarmed RPMI with 5% FBS medium was added to the electroporated *Ehrlichia*. The PNA-*Ehrlichia* suspension was transferred to a T25 flask containing 5 × 10^5^ THP-1 cells for internalization at 37°C for 90 min. The cells were washed to remove uninternalized bacteria, and continuously cultured in fresh medium. Ehrlichial and host mRNA and protein expression were measured respectively at 36 and 48 h post-transfection (pt) of bacteria [post-infection (pi)].

### Reverse transcription quantitative PCR (qRT-PCR)

THP-1 cells infected with Etf-1 or CTL PNA-transfected *E. chaffeensis* were harvested at 36 h pi. Cells were washed once with phosphate buffer saline (PBS; 137 mM NaCl, 2.7 mM KCl, 10 mM Na_2_HPO_4_, 2 mM KH_2_PO_4_, pH 7.4) and total cellular RNA was extracted from ~10^6^ cells per sample using an RNeasy kit (Qiagen, Valencia, CA). RNA concentrations and quality were determined by NanoDrop (Thermo Fisher). Total RNA (2 μg) was reverse transcribed using a Maxima H Minus First Strand cDNA Synthesis Kit and random hexamers (Thermo Fisher). The qPCR reaction mixture (20 μl) included 2 μl cDNA (corresponding to 0.2–0.3 μg of total RNA), 0.25 μM each primer and 10 μl SYBR Green qPCR master mix (Thermo Fisher). PCR was performed in an Mx3000P instrument (Stratagene). Primer sequences are described elsewhere for Etf-1(Liu et al., 2012) and *E. chaffeensis* 16S rRNA, and human G3PDH (Miura et al., 2011).

### Etf-1 protein expression analysis

Host cell-free *E. chaffeensis* were transfected with CTL or Etf-1 PNA and used to infect THP-1 or HEK293 cells. The infected cells were harvested by centrifugation at 48 h pi. The cell pellet was washed with PBS and solubilized in sodium dodecyl sulfate-polyacrylamide gel electrophoresis (SDS-PAGE) sample buffer. Proteins were separated by 10% SDS-PAGE, and transferred to a nitrocellulose membrane (Bio-Rad). Western blot analyses were performed using rabbit anti-Etf-1 (anti-ECH0825) antibody (Liu et al., 2012), rabbit anti-*E. chaffeensis* P28 major outer membrane protein (Ohashi et al., 1998) and rabbit anti-human LC3 (Cell Signaling Technology, Danvers, MA) at 1:1,000. To normalize protein loading, the membrane was washed with Restore ™ Western Blot Stripping buffer (Thermo Fisher) and probed with mouse monoclonal anti-human tubulin (Santa Cruz Biotechnology, Santa Cruz, CA) at 1:1,000. Primary antibodies were detected with horseradish peroxidase-labeled goat anti-rabbit IgG/goat anti-mouse IgG (KPL, Gaithersburg, MD). Blots were developed with Pierce ™ ECL Western Blotting Substrate (Thermo Fisher). Chemiluminescent bands were visualized using an LAS3000 image documentation system (FUJIFILM Medical Systems USA, Stamford, CT) and band intensities were determined by densitometry using Multi Gauge software (FUJIFILM).

### Trans-complementation of *E. chaffeensis* transfected with Etf-1 PNA-transfected and Etf-1-expressing human cells

HEK293 cells at 1 × 10^6^ cells/100 μl were mixed with 5 μg (1 μg/μl) of pEGFPN1 plasmid (Clontech, Mountain View, CA) or pEGFPN1-Etf-1 (pEGFN1-ECH0825 codon-optimized) plasmid (Liu et al., 2012) in a 0.2 cm electroporation cuvette (Bio-Rad). Electroporation was performed using a Gene PulserXcell Microbial System (Bio-Rad) by applying a ~50 ms pulse (100 V, 1,000 μF, ∞ Ω) as described earlier (Niu et al., 2008; Liu et al., 2012). Transfected host cells were grown in DMEM medium (Corning Cellgro) supplemented with 10% FBS and 2 mM L-glutamine in 6-well plate at 1 × 10^6^ cells/well at 37°C in 5% CO_2_ and 95% air in a humidified atmosphere for 24 h. Then host cell-free *E. chaffeensis* transfected with 3 μg of CTL or Etf-1 PNA as described above was added to HEK293 cells that were pre-transfected with pEGFP/pEtf-1-EGFP at approximate MOI of 100:1. The MOI was estimated as follows: GFP- or Etf-1-GFP-transfected uninfected HEK293 cells were cultured at 1 × 10^6^ cells/well for 24 h and the host cell-free bacteria were added; at the time of bacterial addition, number of HEK293 cells were ~2 × 10^6^ cells/well. Host cell-free ehrlichia derived from 4 × 10^7^ infected cells (4 × 10^9^ bacteria) were divided into 4 groups = 1 × 10^9^ bacteria/group; however, after sonication to liberate bacteria, filtration, and electroporation, the rate of final recovery of viable bacteria was ~20%. Infected cells were harvested at 48 h pi (48 h CTL/Etf-1 PNA pt of ehrlichia, and 72 h Etf-1-GFP/GFP pt of HEK293 cells). RNA extraction, cDNA synthesis, qPCR, and western blot analysis were performed as described above.

### Statistical analyses

Experiments were repeated at least three times. Comparison of each treatment was performed relative to samples with CTL PNA. Statistical analysis was performed by 2-tailed Student's *t*-test and *P* < 0.05 was considered significant. For experiments involving more than two groups, analysis of variance was performed and *P* < 0.05 was considered significant. All statistical analyses were performed using Microsoft Excel 2010.

## Results

### *E. chaffeensis* Etf-1 PNA specifically hybridized with its target sequence

Although, the consensus Shine-Dalgarno (SD) sequence is GGAGG, most *E. coli* SD sequences are slight variations of GGAGG (Schurr et al., 1993). The optimal spacing between SD sequence and AUG translational start codon is about 8 to 10 bases for *E. coli* genes (Chen et al., 1994). Ma et al. (2002) reported only 18.6% of *Rickettsia prowazeki* genes has the SD consensus sequence (GGAGG). Similarly, we found most *E. chaffeensis* genes lack the typical SD sequence. Therefore, we assumed 8 to 10 bases upstream of AUG is the putative SD region and designed anti-sense PNA complementary to this region of *E. chaffeensis* Etf-1, and confirmed the PNA sequence specificity by BLASTn analysis against the entire genome of *E. chaffeensis* Arkansas (Dunning Hotopp et al., 2006) (Figure 1A). Scrambled CTL PNA was designed by confirming the absence of annealing sequences by BLASTn analysis against the entire genome of *E. chaffeensis*. To verify that Etf-1-PNA could specifically hybridize with the respective target sequence, the PNA was biotinylated, and biotinylation of PNA was confirmed via dot blot assay (Figure 1B). To demonstrate specific hybridization, equal amounts of the target ssRNA were incubated with biotinylated Etf-1 PNA or different ratios of labeled to non-labeled Etf-1 PNA, and the mixture was analyzed by electrophoresis migration shift assay. Biotinylated Etf-1 PNA showed delayed migration in the presence of the target ssRNA compared to the absence of ssRNA, which indicated hybridization between the target ssRNA and the biotinylated PNA; whereas, without biotinylated PNA, no signal was detected (Figure 1C). Hybridized biotinylated PNA was decreased in a dose-dependent manner in the presence of increasing concentrations of unlabeled Etf-1 PNA, which indicated competitive inhibition of binding (Figure 1C). The formation of ssRNA-PNA pairing established the ability of our Etf-1 PNA to hybridize with the specific nucleotide target sequence.

### PNA treatment impaired ehrlichial Etf-1 expression and infection

**Figure 2:**
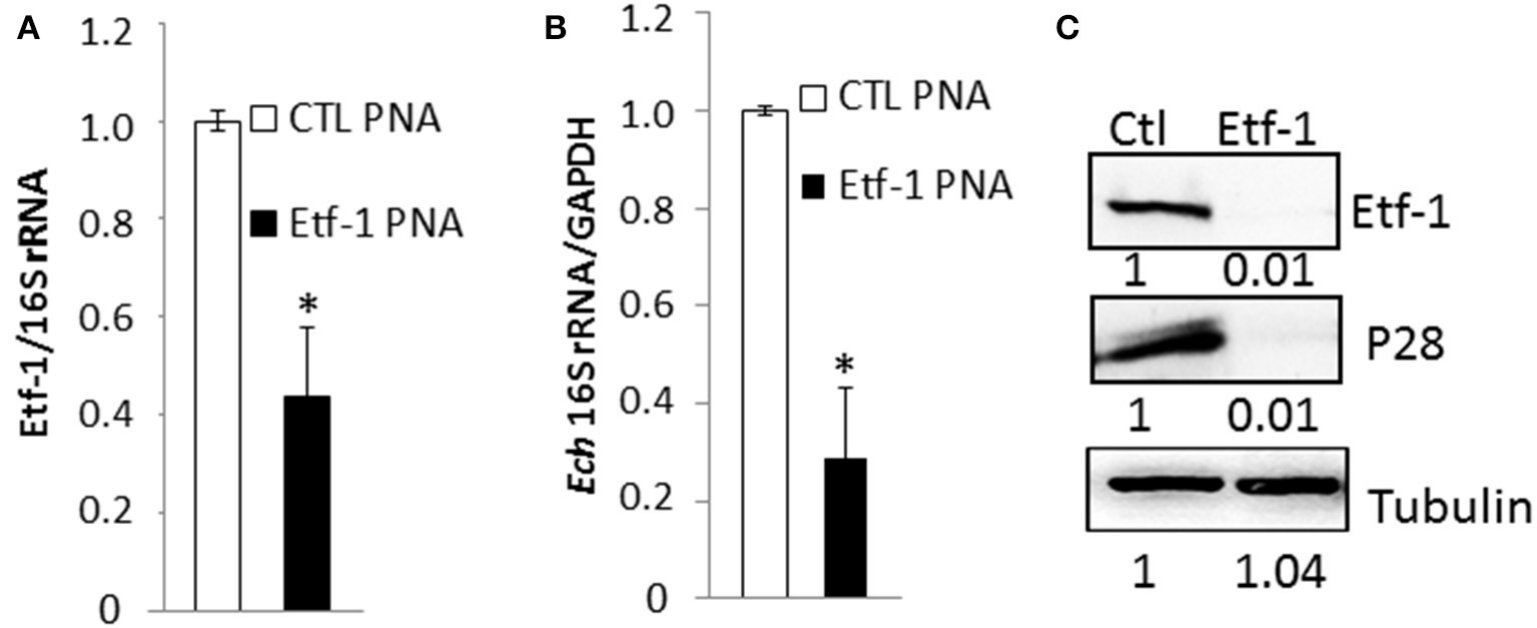
*E. chaffeensis* Etf-1 PNA impaired Etf-1 expression and ehrlichial infection. **(A,B)** Etf-1 PNA inhibited *E. chaffeensis* Etf-1 expression **(A)** and infection **(B)**. RNA samples prepared from THP-1 cells incubated with Etf-1 or CTL PNA-transfected *E. chaffeensis* were harvested at 36 h pi and subjected to qRT-PCR analysis. The values reflect bacterial 16S rRNA normalized against human GAPDH mRNA, relative to the amount determined for the CTL PNA treatment. Data indicate the mean ± standard deviation from three independent experiments performed in triplicate. ^*^Significantly different (*P* < 0.05; Student's *t*-test) compared to the value of the CTL PNA. **(C)** Western blot analysis of Etf-1 and *E. chaffeensis* outer membrane protein P28 normalized against human tubulin in THP-1 cells incubated with Etf-1 and CTL PNA-transfected *E. chaffeensis* at 48 h pi. The ratios of Etf-1: tubulin and p28:tubulin were quantified and compared to CTL PNA, which was set to 1.

Knowing that the PNA was specific for its target gene, we evaluated ability of Etf-1 PNA to suppress Etf-1 mRNA expression in ehrlichiae and inhibit its infection of human monocytes. *E. chaffeensis* was purified to homogeneity, electroporated with non-targeting CTL or Etf-1 PNA, and incubated with the human monocyte cell line THP-1. Etf-1 mRNA and *E. chaffeensis* 16S rRNA (to measure live bacteria) levels were analyzed by qRT-PCR. Human GAPDH mRNA levels (to measure live host cells) were also analyzed to normalize the sample. Etf-1 mRNA/16S rRNA in *E. chaffeensis* and 16S rRNA/human GAPDH mRNA in host cells were significantly reduced at 36 h pt (pi) compared to CTL PNA-treated *E. chaffeensis* (Figures 2A,B). Western blot analysis revealed profound reduction of *E. chaffeensis* P28 major outer membrane proteins (to measure bacteria at protein level; encoded by a multigene family and major two bands in human cells) (Ohashi et al., 1998) and Etf-1/human tubulin (to normalize host cells at protein level) in Etf-1 PNA-treated *E. chaffeensis* at 48 h pt (pi) compared with CTL PNA-treated *E. chaffeensis* in THP-1 cells (Figure 2C). The result indicated that Etf-1 mRNA and protein expression was effectively knocked down with the Etf-1 PNA, Etf-1 was required for *E. chaffeensis* infection, and thus, the PNA approach was useful for examining the requirement of target ehrlichial genes in infection.

### Reduced ehrlichial growth by Etf-1 PNA knockdown was complemented with ectopically expressed Etf-1-GFP

**Figure 3:**
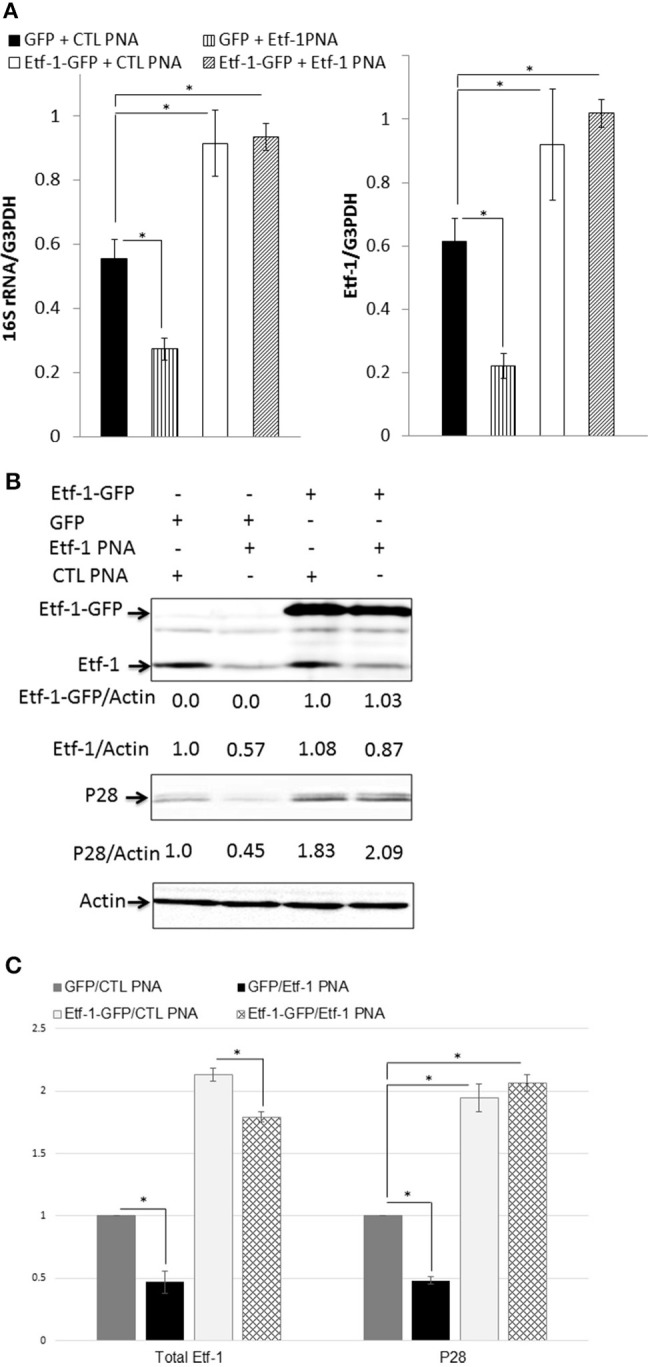
Intracellular complementation of Etf-1 PNA-reduced ehrlichial infection by ectopic expression of Etf-1-GFP. HEK293 cells expressing Etf-1 complemented the Etf-1 PNA knockdown and rescued *E. chaffeensis* infection. RNA samples prepared from HEK293 cells expressing Etf-1-GFP or GFP incubated with Etf-1 or CTL PNA-transfected *E. chaffeensis* were harvested at 48 h pi and subjected to qRT-PCR analysis **(A)** and protein analysis **(B,C)**. The values reflect bacterial 16S rRNA and total Etf-1 normalized against human GAPDH mRNA, relative to the amount determined for the Etf-1 PNA treatment of HEK293 cells expressing Etf-1-GFP. Data indicate the mean ± standard deviation from three independent experiments performed in triplicate. ^*^Significantly different (*P* < 0.05; analysis of variance) **(A)**. Western blot analysis using anti-P28, actin, and Etf-1. The values under the bands show relative ratios of band intensities vs. actin, with the ratios of those from GFP plasmid and/or CTL PNA set to 1. **(B)** Results are presented as means ± standard deviations from three independent experiments. The asterisk indicates a significant difference compared with CTLs by analysis of variance (*P* < 0.01).

To rule out the possibility that the observed phenotypes of *E. chaffeensis* were caused by disruption of off-target sites, we performed an intracellular rescue assay by ectopic expression of Etf-1-GFP in host cells. To negate the effect of foreign plasmid, as control, host cells were transfected with GFP plasmid. Because THP-1 cells are difficult to transfect with plasmids, we used HEK293 cells that can be easily transfected and infected with *E. chaffeensis* (Miura et al., 2011). Our previous studies showed that *E. chaffeensis* behaved similarly in human monocytes and HEK293 cells (Liu et al., 2012; Lin et al., 2016). HEK293 cells were transfected with Etf-1-GFP or GFP plasmid. At 24 h pt, i.e., the time point when Etf-1-GFP expression and Etf-1-GFP-induced autophagy is clearly visible in the host cells (Lin et al., 2016), Etf-1 PNA or CTL PNA-treated *E. chaffeensis* were added to the pre-transfected HEK293 cells and cells were harvested at 48 h pi. The results showed that ehrlichial Etf-1 mRNA/human GAPDH mRNA and *E. chaffeensis* 16S rRNA/human GAPDH mRNA were significantly reduced in Etf-1 PNA than in CTL PNA-treated *E. chaffeensis* in HEK293 cells (Figure 3A). As we previously reported with non-PNA-treated *E. chaffeensis* (Lin et al., 2016), based on the levels of *E. chaffeensis* 16S rRNA/human GAPDH mRNA, Etf-1-GFP-transfection of HEK293 cells enhanced infection of CTL PNA-treated *E. chaffeensis* compared to the GFP-transfection CTL (Figure 3A). Etf-1-GFP-transfection of HEK293 cells enhanced infection by Etf-1 PNA-treated *E. chaffeensis* compared to the GFP-transfection CTL as well (Figure 3A). At protein level, western blot analysis revealed significant reduction of ehrlichial Etf-1 and reduction of *E. chaffeensis* P28 major outer membrane proteins with Etf-1 PNA-treated *E. chaffeensis* at 48 h pi compared with CTL PNA-treated *E. chaffeensis* in HEK293 cells (GFP-transfection CTL in Figures 3B,C). Etf-1-GFP transfection of HEK 293 cells resulted in a significant increase in ehrlichial Etf-1 and P28 protein levels compared to GFP-transfection CTL, in both Etf-1 PNA and CTL PNA-treated *E. chaffeensis* (Figures 3B,C). Taken together, these data indicate that exogenous Etf-1-GFP expression intracellularly complemented Etf-1 PNA-mediated inhibition of ehrlichial Etf-1 expression and *E. chaffeensis* infection.

### Etf-1 PNA knockdown impairs *E. chaffeensis*-induced host cell autophagy

**Figure 4:**
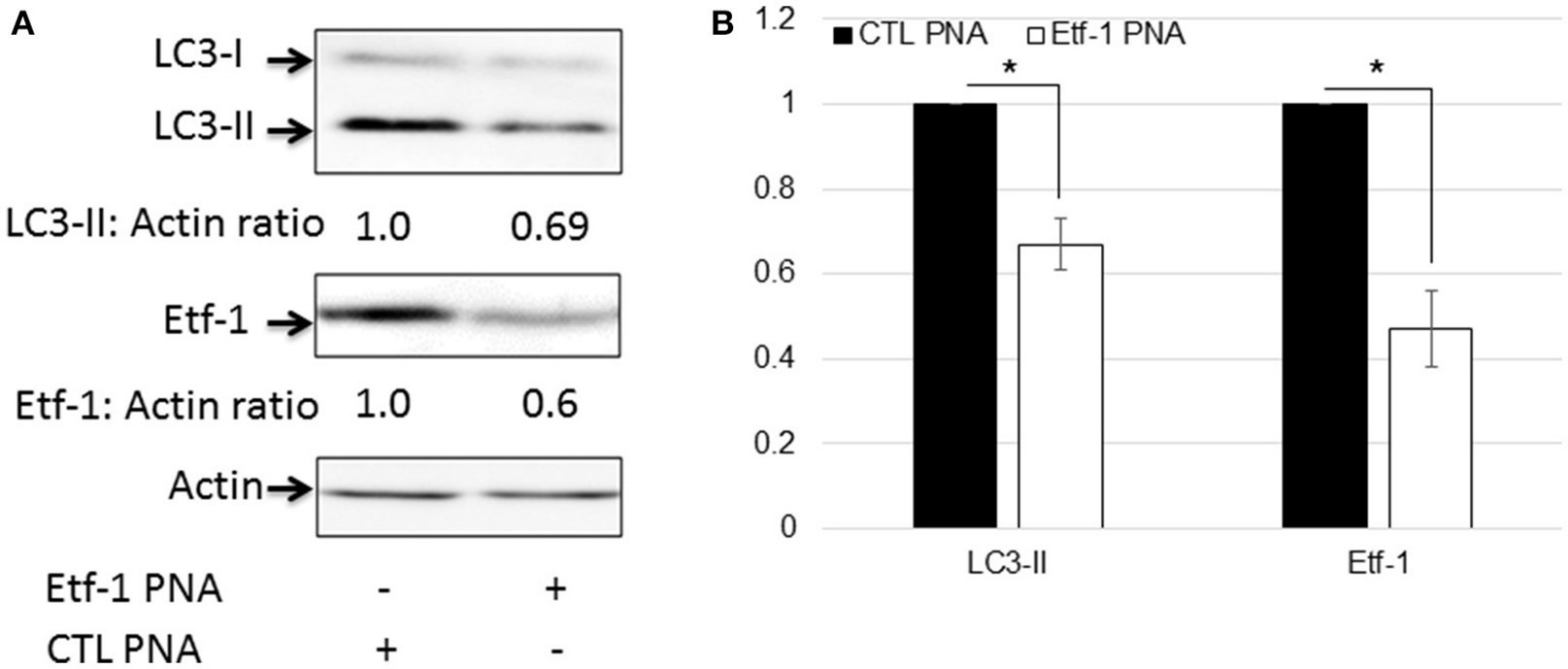
Etf-1 PNA knockdown impedes *E. chaffeensis*-induced host cell autophagy. HEK293 cells were infected with Etf-1 or CTL PNA-transfected *E. chaffeensis* and harvested at 48 h pi and subjected to western blot analysis using anti-LC3, actin, and Etf-1 **(A,B)**. The values under the bands show relative ratios of band intensities vs. actin, with the ratios of those from CTL PNA set to 1. **(B)** Results are presented as means ± standard deviations from three independent experiments. The asterisk indicates a significant difference compared with CTLs by Student's *t*-test (*P* < 0.01).

Autophagy is a catabolic process for the autophagosome-lysosomal degradation of bulk cytoplasmic contents (Yorimitsu and Klionsky, 2005; Klionsky, 2007). Autophagy is an important innate immune mechanism to clear intracellular infection (Deretic, 2011; Levine et al., 2011). However, intracellular *E. chaffeensis* is not only resistant to host cell autophagy, but rather ehrlichial infection is enhanced by host cell autophagy, and ehrlichial Etf-1 secreted into host cell cytoplasm or ectopically expressed Etf-1-GFP induces host cell autophagy and promote ehrlichial growth (Lin et al., 2016). Mammalian light Chain 3 (LC3, MAP1LC3, the homolog of yeast ATG8) undergoes post-translational modifications during autophagy: the cytosolic form LC3-I is converted to LC3-II through cleavage and lipidation that allows LC3 to become associated with autophagic vesicles (Kabeya et al., 2000). The presence of LC3 in autophagosomes and the conversion of LC3-I to the lower migrating form LC3-II have been used as indicators of autophagy (Klionsky et al., 2008). To investigate whether Etf-1 PNA treatment of *E. chaffeensis* inhibited ehrlichial Etf-1-induced cellular autophagy, HEK293 cells were infected with Etf-1 PNA or CTL PNA-treated *E. chaffeensis* and the cells were harvested at 48 h pi. Western blot analysis revealed significant reduction of ehrlichial Etf-1 protein expression, and decrease in LC3-II levels with Etf-1 PNA-treated *E. chaffeensis* at 48 h pi compared with CTL PNA-treated *E. chaffeensis* in HEK293 cells (Figures 4A,B). Taken together, these data indicate that Etf-1 PNA treatment reduces ehrlichial Etf-1 levels and consequently diminishes Etf-1-mediated host cell autophagy induced by *E. chaffeensis* infection.

## Discussion

Significant progresses have been made in generating mutants in rickettsial organisms including *Rickettsia, Anaplasma*, and *Ehrlichia* by random transposon mutagenesis and by transformation with rickettsial or other plasmids, and these procedures generated stable florescent bacteria that allows to track bacterial growth and motility, and study *in vitro* and *in vivo* functions of knockout mutants isolated using the cell culture system (Felsheim et al., 2006, 2010; Kleba et al., 2010; Noh et al., 2011; Chen et al., 2012; Cheng et al., 2013; Crosby et al., 2014; Oliver et al., 2014; Oliva Chavez et al., 2015; Hauptmann et al., 2017). However, so far stable targeted mutagenesis including conditional mutagenesis that allows functional studies have not been achieved. This also precludes complementation studies using the knockout mutants. One of unique features of obligatory intracellular bacteria is that they have evolved genes essential for survival and proliferation within the hostile eukaryotic cell environment. However, unlike facultative intracellular bacteria, knockout mutants for genes required for intracellular survival cannot be obtained for obligatory intracellular bacteria. Furthermore, obligatory intracellular bacteria have evolved unique genes (e.g., ~50% of *E. chaffeensis* ORFs encode “hypothetical proteins,” which have no homology to any other known proteins (Dunning Hotopp et al., 2006; Rikihisa, 2015), which preclude studies based on conserved motifs and orthologous genes in other bacteria. Indeed, many of novel *Ehrlichia* and *Anaplasma* genes so far discovered as critical for infection encode previously “hypothetical proteins” (Rikihisa, 2010, 2011, 2015). In addition, surrogate systems in model bacteria such as *E. coli* do not provide needed key information on obligatory intracellular survival/infection. When *E. chaffeensis* was treated with PNA specific to Etf-1, host cell autophagy induction and *E. chaffeensis* infection were significantly reduced, which validated the critical role of the T4SS effector for autophagy induction and *E. chaffeensis* infection. Our study demonstrated the utility of PNA delivery as an effective method to study *E. chaffeensis* gene function, which is more broadly applicable for obligatory intracellular bacteria to overcome limited availability of genetictools.

Off target effects are a concern when working with antisense oligos to interrupt gene function. However, advantages of PNAs are that (1) both affinity and specificity of a PNA/DNA duplex is higher than a DNA/DNA duplex (Nielsen and Egholm, 1999), and (2) a mismatch in a PNA/DNA duplex is generally more destabilizing than a mismatch in a DNA/DNA duplex (Nielsen and Egholm, 1999), thus less likely causes knockdown of the mismatched gene. When designing PNA oligos, we blasted the PNA sequences against the entire *E. chaffeensis* genome sequence to ensure alignment with only our target sequence: Etf-1 PNA designed complementary to the putative SD region. To negate the non-specific effect of PNA transfection in *Ehrlichia*, all the experiments were performed in parallel with scrambled CTL PNA. Our competitive hybridization assay results with ssRNA, cell culture infections, and mRNA and protein suppression experiments pointed to the specificity of the PNA approach for the target. Thus, appropriately designed PNA, well-controlled experiments, and objective evaluation as used in the present study (qRT-PCR and densitometry analysis of protein expression) would be expected to facilitate the discovery of unique genes important for obligatory intracellular bacteria. Our study showed that, for secreted target proteins such as Etf-1, deficiency can be complemented by ectopic expression of Etf-1 in the host cells, offering a new approach to affirm the specificity of PNA knockdown. One of the limitations of the PNA approach is the cost of custom PNA synthesis. Although, almost all published papers on PNA use a cell-permeable peptide (CPP) linked to the N-terminus of the PNA, for our study without CPP, PNA was effectively delivered into infectious live *Ehrlichia*, which reduced the cost of PNA synthesis.

The field needs additional tools of genetic manipulation to better scrutinize the lifestyle and virulence of obligate intracellular bacteria, and allow molecular Koch's postulates to be fulfilled. Our ability to inhibit protein expression in pathogenic *E. chaffeensis* indicates the broad spectrum of uses for this technology. PNA offers an advantage in that reduction of protein expression is achieved in 2 days, no antibiotic selection is required, and partial reduction can be achieved allowing study of essential genes, where investigations of a phenotype imposed by a true knockout would be impossible due to bacterial death. Given that anti-sense oligos have been developed as FDA-approved drugs for certain diseases (Jiang, 2013), by modifying PNAs to improve their targeting of specific mammalian cell types and intracellular bacteria, it is reasonable to envision enhanced bioavailability *in vivo*. The work presented herein represents a necessary first step in the path of developing PNAs as a drug to effectively block *E. chaffeensis* infection.

## Author contributions

Conceived and designed the experiments: YR. Performed experiments and analyzed data: PS, OT, and YR. Wrote the paper: YR and PS.

### Conflict of interest statement

The authors declare that the research was conducted in the absence of any commercial or financial relationships that could be construed as a potential conflict of interest.
